# Ocular inoculation of toad venom: toxic cataract and proteomic profiling

**DOI:** 10.3389/fmed.2024.1537770

**Published:** 2025-01-14

**Authors:** Danni Lyu, Shuang Ni, Jia Xu, Sha Zhu, Jing-Wei Xu, Yixuan Feng, Ce Shi, Wen Xu

**Affiliations:** ^1^Eye Center of the 2nd Affiliated Hospital, School of Medicine, Zhejiang University, Hangzhou, Zhejiang, China; ^2^Zhejiang Provincial Key Lab of Ophthalmology, Hangzhou, Zhejiang, China

**Keywords:** toad venom, venom inoculation, toxic cataract, 4D label-free quantitative proteomics, Na^+^/K^+^-ATPase

## Abstract

**Purpose:**

To report a singular case of cataract caused by toad venom inoculation and to scrutinize the pathological mechanisms through proteomic sequencing of the lens specimen.

**Methods:**

A young Chinese male presented with progressively deteriorating vision in his right eye subsequent to a history of toad venom inoculation. He was diagnosed with a toxic cataract, and underwent phacoemulsification cataract surgery. Anterior capsule, nucleus, and cortex specimens from the patient (designated as PT_CAP, PT_PHACO, and PT_CTX, respectively) and age-related cataract controls (C_CAP, C_PHACO, and C_CTX, respectively) were collected and subjected to 4D label-free quantitative proteomics.

**Results:**

A multitude of differentially expressed proteins (DEPs) were identified in the patient’s lens compared to those in the controls. Specifically, a total of 204 DEPs were identified in PT_CAP compared to C_CAP, with MYH6, MYL2, MYL3, STAT1, and ANK1 among the foremost regulated DEPs. The DEPs of PT_CAP were principally affiliated with functions including “transportation of small molecules,” “regulation of metal ion transport,” and “import into cell.” A sum of 109 DEPs were delineated in PT_CTX compared to C_CTX, with TPM1 among the top-10 downregulated DEPs. Ninety-five DEPs were pinpointed in PT_PHACO compared to C_PHACO, with hexokinase among the top 10 downregulated DEPs. These proteins were ascertained to be linked with Na^+^/K^+^-ATPase activity.

**Conclusion:**

This study introduced the first documented case of toxic cataract caused by toad venom inoculation. Proteomic sequencing indicated a correlation between cataract and alterations in Na^+^/K^+^-ATPase activity, providing insights for the clinical management of ocular toad venom inoculation in subsequent cases.

## Introduction

Toad venom represents a frequently encountered animal toxin capable of inducing both local and systemic toxicity. Comprised of bufadienolides synthesized by the parotid glands and skin, it serves as a potential inhibitor of the sodium-potassium adenosine triphosphatase (Na^+^/K^+^-ATPase) pump. Bufotoxin, a principal toxic component, exhibits effects akin to those of digitalis (1). Systemic manifestations of toad venom poisoning encompass cyanosis, paralysis, seizures, increased salivation, vomiting, hyperkalemia, and hallucinations (1). Direct ocular exposure to toad venom may incite acute ocular toxicity, termed toad venom inoculation, manifesting as clinical symptoms and signs such as chemosis, conjunctivitis, keratitis, stromal corneal edema with Descemet’s folds, corneal dysfunction, and ocular hypotonia (1–3). In severe cases, local ocular mucosal absorption can precipitate systemic responses such as hypertension, bradycardia, and arterial constriction (4). Nonetheless, reports pertaining to the long-term ocular toxicity of toad venom remain scarce.

The present article elucidated a distinctive case of cataract resulting from toad venom exposure in our clinical observation. We conducted proteomic sequencing of the lens specimen from the affected eye to explore alterations in protein expression across various portions of the lens induced by toad venom. Our objective was to delineate the pathological mechanisms underlying cataracts triggered by toad venom inoculation and to establish a foundation for future treatment approaches for ocular toad venom exposure.

## Case report

**Figure 1:**
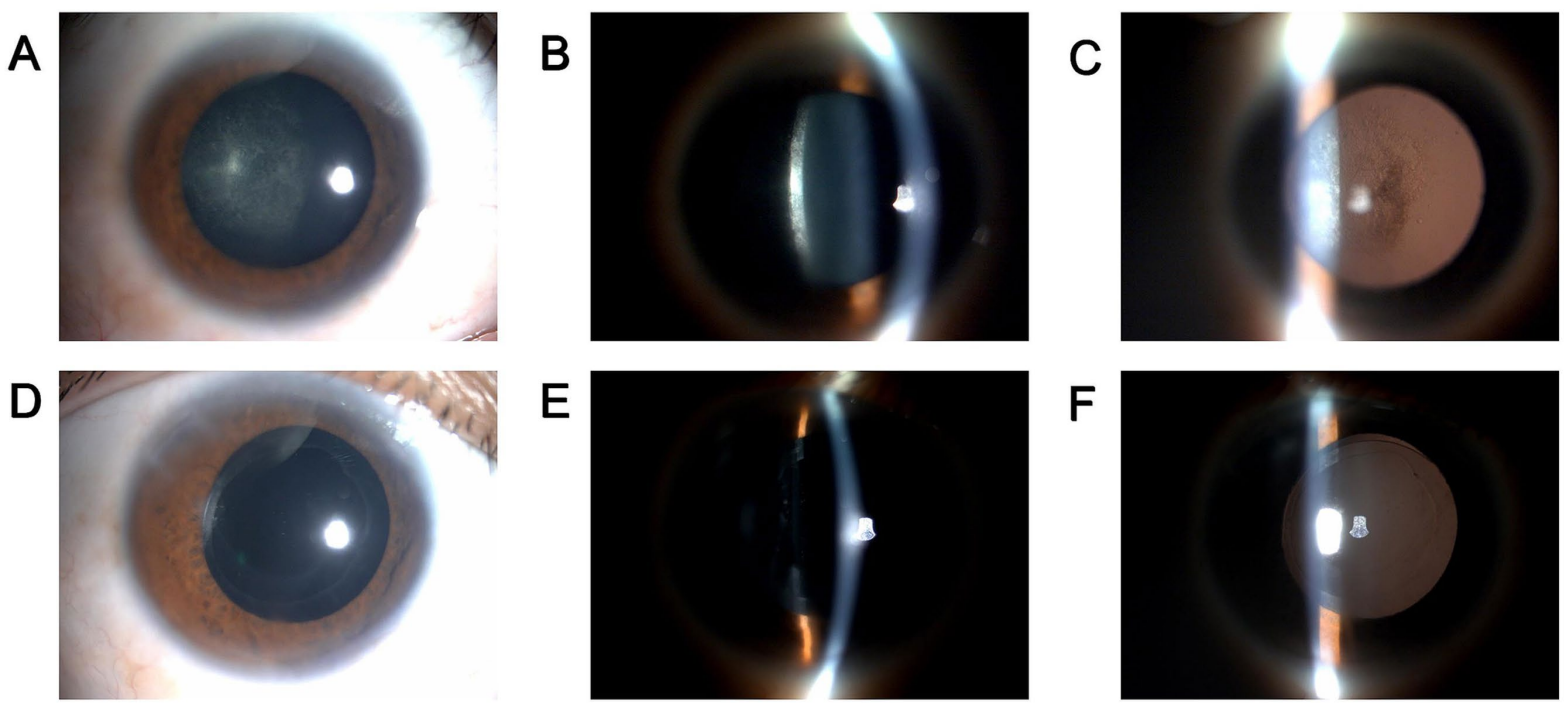
Preoperative and postoperative clinical data of the patient. **(A–C)** Slit lamp photography of the patient’s right eye before surgery. **(A)** diffuse illumination; **(B)** direct focal illumination; **(C)** retro illumination. **(D–F)** slit lamp photography of the patient’s right eye at 1 week after surgery. **(D)** diffuse illumination; **(E)** direct focal illumination; **(F)** retro illumination.

A 29-year-old Chinese male, henceforth referred to as the patient, presented to our ophthalmology clinic reporting progressively deteriorating vision in his right eye over a span of 2 years. The patient recounted a prior incident wherein toad venom had inadvertently splashed into his right eye, resulting in transient symptoms of redness and irritation. Upon examination, his best-corrected distance visual acuity (BCDVA) was 5/20 in the right eye and 20/20 in the left eye. Slit lamp microscopy evaluation of the right eye revealed noticeable posterior subcapsular opacity (PCO), graded as C_1_N_0_P_4_ according to the LOCS II classification (Figures 1A–C). Subsequent ocular assessments did not reveal any significant abnormalities. A diagnosis of toxic cataract in the right eye was established.

The patient underwent standard micro-incision phacoemulsification and intraocular lens (IOL) implantation procedures under topical anesthesia. A sample of the anterior capsule was collected, and the designation PT_CAP was assigned. Phacoemulsification was employed to remove the lens nucleus, and the resulting phacoemulsification fluid was collected and labeled as PT_PHACO, representing the nucleus sample given that the phacoemulsification procedure predominantly targets the nucleus. Subsequent manual irrigation and aspiration techniques were utilized to extract the residual lens cortex, with the collected sample designated as PT_CTX.

During the 1-week postoperative follow-up, clinical evaluation of the operated eye revealed a transparent cornea, a tranquil anterior chamber, and appropriately positioned IOL (Figures 1D–F). The BCDVA in the right eye improved to 20/25. Subsequent ocular examinations did not reveal any noteworthy findings. Owing to the epidemic circumstances, subsequent follow-up visits at 1 month, 3 months, 6 months, and 12 months were conducted at the local eye hospital under our team’s supervision, yielding satisfactory outcomes (details not provided).

## Materials and methods

### Collection of the clinical data

The study protocol strictly adhered to the ethical principles delineated in the Declaration of Helsinki and obtained approval from the Medical Ethics Committee of the Second Affiliated Hospital of Zhejiang University School of Medicine, Hangzhou, China. The patient underwent standard preoperative and postoperative evaluations, as elaborated in the Supplementary materials. All clinical data were meticulously retrieved from the electronic medical record system, with the patient’s explicit written consent duly obtained.

### Surgical procedure and sample collection

The surgical procedure details and sample collection for the patient are meticulously delineated in the Case Report section. Additionally, three patients afflicted with age-related cataracts but devoid of systemic diseases were recruited as the control cohort (hereafter referred to as controls). Employing a standardized protocol identical to that of the patient, regular micro-incision phacoemulsification and IOL implantation procedures were administered to the controls. Samples of the lens anterior capsule, phacoemulsification collection fluid, and lens cortex were collected and designated as follows: Control’s lens anterior capsule samples 1, 2, and 3 (C_CAP 1, C_CAP 2, and C_CAP 3), Control’s phacoemulsification collection fluid samples 1, 2, and 3 (C_PHACO 1, C_PHACO 2, and C_PHACO 3), and Control’s lens cortex samples 1, 2, and 3 (C_CTX 1, C_CTX 2, and C_CTX 3), respectively. The procurement of specimens from both the patient and donors was conducted subsequent to the acquisition of written informed consent.

### 4D label-free quantitative proteomic analysis

Refer to the Supplementary materials and Methods section for detailed information.

## Results

### Proteomic analysis of patient’s lens versus controls

**Figure 2:**
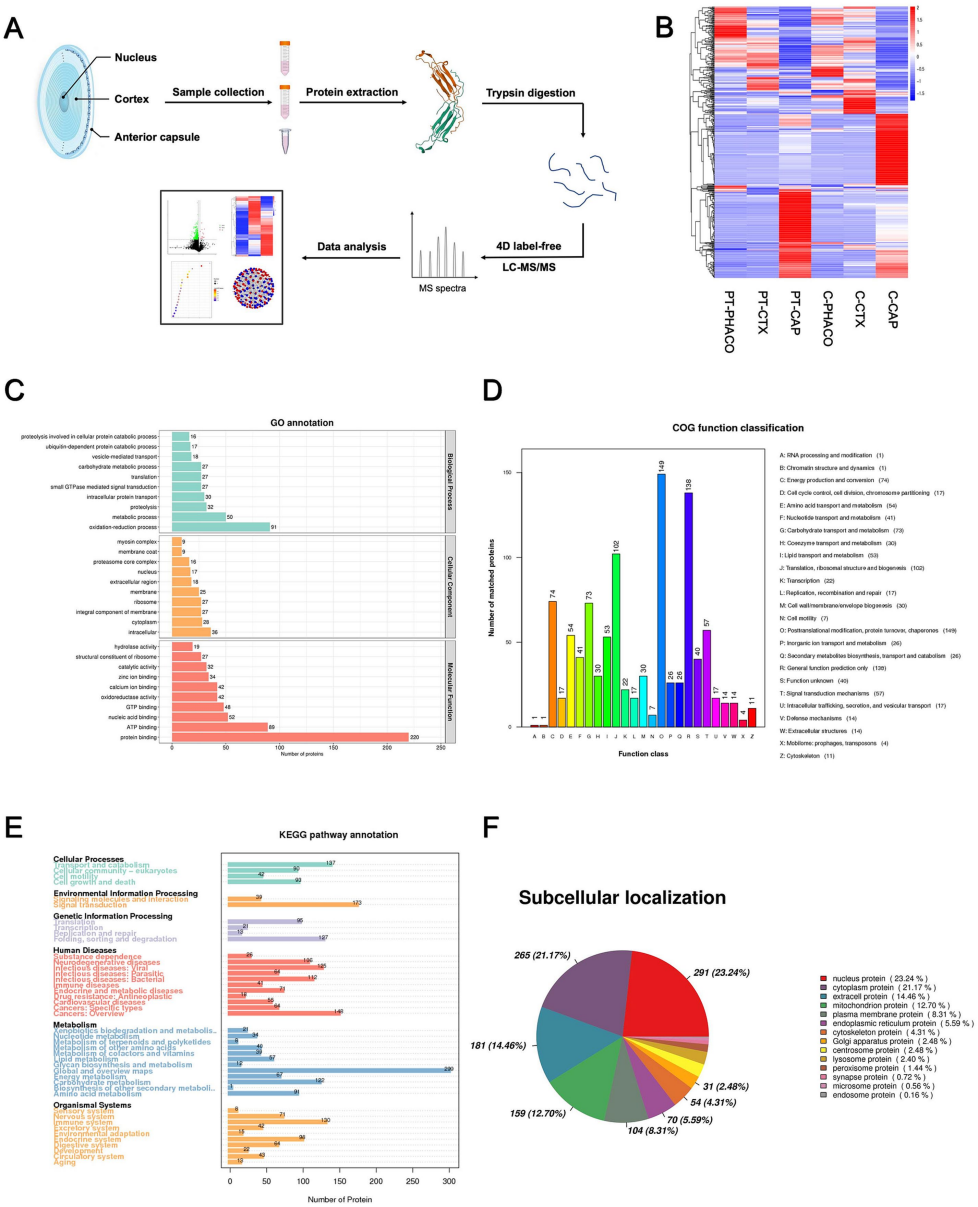
4D label-free quantitative proteomics analysis of DEPs among all the different groups. **(A)** Flow diagram of 4D label-free quantitative proteomics analysis. **(B)** Heatmap generated from a hierarchical cluster analysis, illustrating the different expression profiling between patient’s and controls’ lens specimens. PT_PHACO, patient’s phacoemulsification collect fluid; PT_CTX, patient’s cortex sample; PT_CAP, patient’s anterior capsule sample; C_PHACO, controls’ phacoemulsification collect fluid; C_CTX, controls’ cortex sample; C_CAP, controls’ anterior capsule sample. **(C)** GO classification of the DEPs in terms of BP, CC, and MF among all the groups. **(D)** COG function classification of the DEPs among all the groups. **(E)** Enrichment of KEGG pathways among all the groups. **(F)** Subcellular localization of the DEPs among all the groups.

Protein extracts originating from distinct components of both the patient’s and controls’ lenses were meticulously collected for 4D Label-Free analysis, as depicted in the flow diagram presented in Figure 2A. Numerous differentially expressed proteins (DEPs) were discerned in the anterior capsule, cortex, and nucleus of the patient’s lens relative to those in the controls (Figure 2B; Supplementary Table S2). Subsequent Gene Ontology (GO), Clusters of Orthologous Groups (COG) function, and Kyoto Encyclopedia of Genes and Genomes (KEGG) pathway analyses were conducted based on all DEPs. The GO categories encompassing biological process (BP), cellular component (CC), and molecular function (MF) are depicted in Figure 2C, elucidating that “oxidation–reduction process,” “intracellular,” and “protein binding” emerged as the most prevalent terms among the BP, CC, and MF categories, respectively. COG and KEGG categories are delineated in Figures 2D,E, respectively. Additionally, a subcellular localization analysis of the DEPs was undertaken, as delineated in Figure 2F.

### Proteomic analysis of anterior capsule of patient’s lens versus controls’ lens

**Figure 3:**
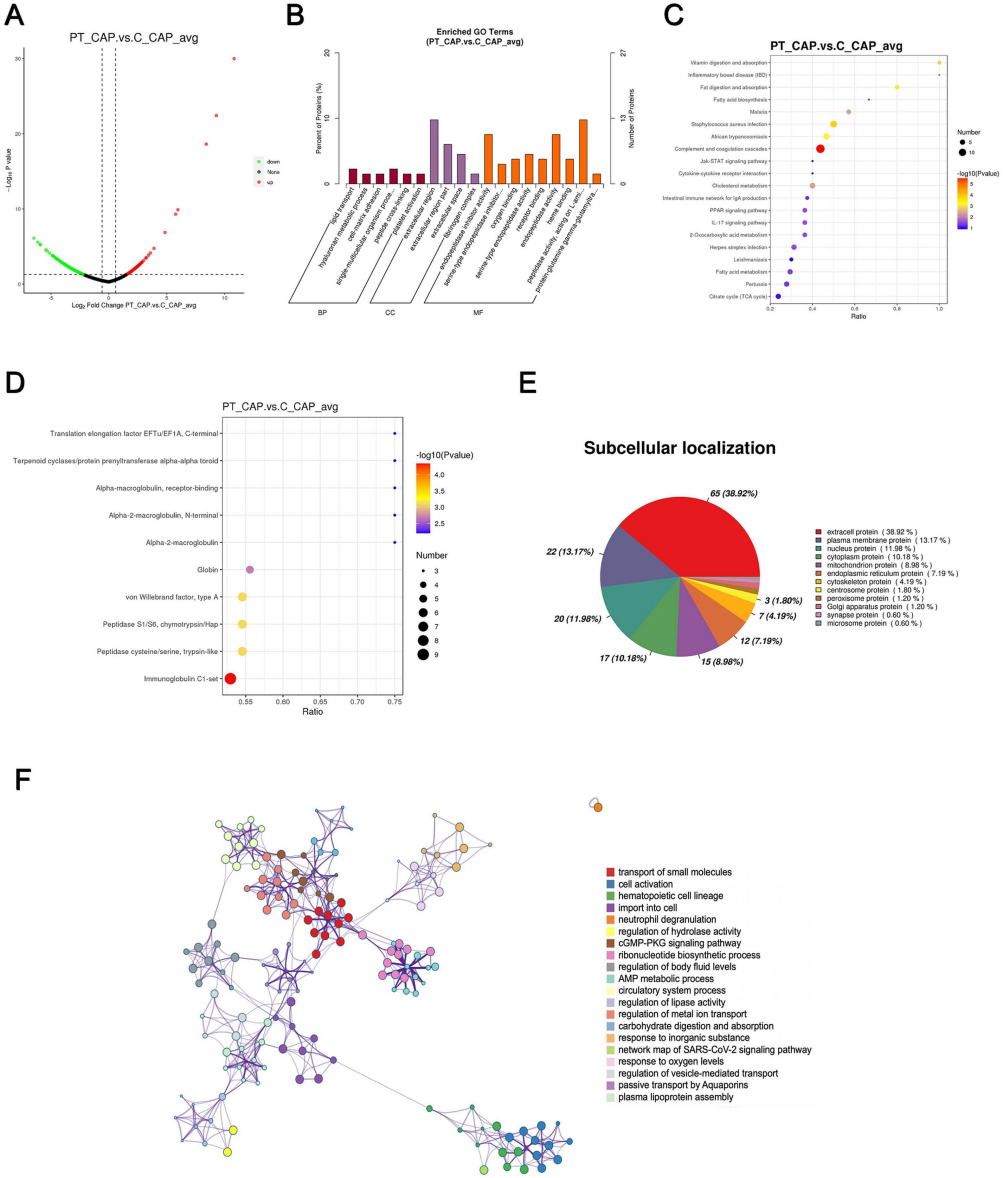
Quantitative proteomics analysis of DEPs between the patient’s anterior capsule sample (PT_CAP) and the controls’ anterior capsule specimens (C_CAP). **(A)** Volcano plot of DEPs. **(B)** GO classification of the DEPs between PT_CAP and C_CAP in terms of BP, CC, and MF. **(C)** Enrichment of KEGG pathways between PT_CAP and C_CAP. **(D)** IPR functional analysis of the DEPs between PT_CAP and C_CAP. **(E)** Subcellular localization of the DEPs between PT_CAP and C_CAP. **(F)** PPI network of DEPs between PT_CAP and C_CAP.

In total, a count of 204 DEPs was recorded, comprising 92 upregulated and 112 downregulated proteins in the patient’s anterior capsule in comparison to controls, as delineated in the volcano plot (Figure 3A). The top-10 upregulated and downregulated DEPs are itemized in Tables 1, 2. The identified proteins were categorized in terms of cellular components based on GO functional analysis, with the DEPs cataloged into 19 GO terms encompassing six BPs, four CCs, and nine MFs (Figure 3B). Notably, “extracellular region” emerged as the most abundant term among the CCs, while “peptidase activity, acting on L-amino acid peptides,” “endopeptidase inhibitor activity,” and “endopeptidase activity” ranked among the top three terms within the MF category. Subsequently, KEGG enrichment analysis was conducted to explore the roles of DEPs in the anterior capsule and their impact on lens opacification (Figure 3C). The outcomes unveiled that the pathways exhibited the highest enrichment in “complement and coagulation cascades.” Moreover, InterPro (IPR) enrichment analysis was undertaken to prognosticate the functional classification of the DEPs through domain annotation, revealing that the DEPs demonstrated the highest enrichment in “immunoglobulin C1-set” (Figure 3D). Lastly, subcellular localization prediction of DEPs was executed, with the majority of proteins localized in the extracellular region (38.92%), followed by the plasma membrane (13.17%), and the nucleus (11.98%) (Figure 3E).

**Table 1:** Top 10 up-regulated differentially expressed proteins of PT_CAP compared to C_CAP.

Protein accession	Protein description	Gene name	Fold change	Regulation type
P13533	Myosin-6	MYH6	1802.689604	Up
A0A590UJU8	Myosin regulatory light chain 2, ventricular/cardiac muscle isoform	MYL2	623.1301682	Up
A0A024R2Q5	Myosin, light polypeptide 3, alkali ventricular, skeletal, slow, isoform CRA_a	MYL3	340.6214258	Up
A0A384MDM4	Epididymis secretory sperm binding protein		62.45444494	Up
Q13584	Isocitrate dehydrogenase [NADP]		54.38418039	Up
P42224	Signal transducer and activator of transcription 1-alpha/beta	STAT1	28.87224261	Up
A0A0A0MSV6	Complement C1q subcomponent subunit B (Fragment)	C1QB	14.92037878	Up
A0A087X0S5	Collagen alpha-1(VI) chain	COL6A1	11.99917337	Up
B2R5U3	EH-domain containing 1, isoform CRA_b	EHD1	10.68257526	Up
Q99798	Aconitate hydratase, mitochondrial	ACO2	9.334540919	Up

PT_CAP, patient’s anterior capsule sample; C_CAP, controls’ anterior capsule sample.

**Table 2:** Top 10 down-regulated differentially expressed proteins of PT_CAP compared to C_CAP.

Protein accession	Protein description	Gene name	Fold change	Regulation type
A0A140VK00	Testicular tissue protein Li 227		88.55158998	Down
X6R8F3	Neutrophil gelatinase-associated lipocalin	LCN2	69.98356576	Down
P01833	Polymeric immunoglobulin receptor	PIGR	60.82022521	Down
A0A024R8D7	Lipocalin 1 (Tear prealbumin), isoform CRA_a	LCN1	60.29155792	Down
P12273	Prolactin-inducible protein	PIP	43.33423859	Down
C0JYY2	Apolipoprotein B (Including Ag(X) antigen)	APOB	40.14276695	Down
P16157	Ankyrin-1	ANK1	34.56247160	Down
B2RMS9	Inter-alpha (Globulin) inhibitor H4	ITIH4	29.06215468	Down
A0A024R6P0	Serpin peptidase inhibitor, clade A (Alpha-1 antiproteinase, antitrypsin), member 3, isoform CRA_c	SERPINA3	28.75330803	Down
O75556	Mammaglobin-B	SCGB2A1	28.31796819	Down

PT_CAP, patient’s anterior capsule sample; C_CAP, controls’ anterior capsule sample.

The protein–protein interaction (PPI) network analysis was employed to elucidate the functional interactions among DEPs. As depicted in Figure 3F, the enriched DEP clusters encompassed various functional categories including “transportation of small molecules,” “regulation of metal ion transport,” “circulatory system process,” “ribonucleotide biosynthetic process,” and others. Furthermore, molecular complex detection (MCODE) clusters were utilized to identify densely connected network components, leading to the identification of 19 highly interconnected clusters (Supplementary Figure S1).

### Proteomic analysis of the cortex of the patient’s lens compared to the controls’ lens

**Figure 4:**
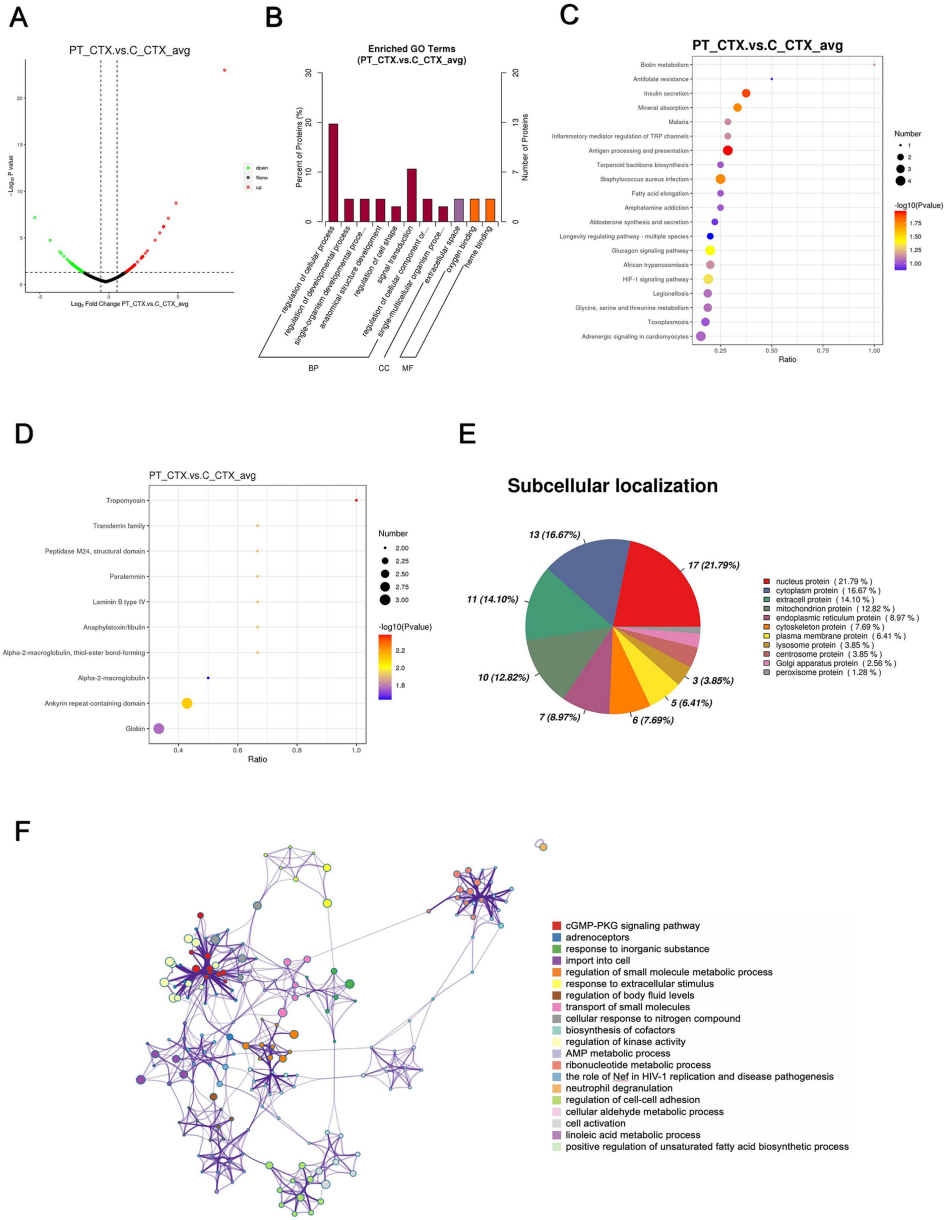
Quantitative proteomics analysis of DEPs between the patient’s cortex sample (PT_CTX) and the controls’ cortex specimens (C_CTX). **(A)** Volcano plot of DEPs. **(B)** GO classification of the DEPs between PT_CTX and C_CTX in terms of BP, CC, and MF. **(C)** Enrichment of KEGG pathways between PT_CTX and C_CTX. **(D)** IPR functional analysis of the DEPs between PT_CTX and C_CTX. **(E)** Subcellular localization of the DEPs between PT_CTX and C_CTX. **(F)** PPI network of DEPs between PT_CTX and C_CTX.

In total, a count of 109 DEPs, comprising 53 upregulated and 56 downregulated proteins, were observed in the patient’s cortex relative to controls, as illustrated in the volcano plot (Figure 4A). The top-10 upregulated and downregulated DEPs are itemized in Tables 3, 4. GO functional classification (Figure 4B) indicated that the DEPs were categorized into 11 GO terms, including eight BP, one CC, and two MFs. Noteworthy terms within the BPs included “regulation of cellular process” and “signal transduction.” The top-10 KEGG categories are presented in Figure 4C, revealing enrichment in pathways such as “antigen processing and presentation,” “insulin secretion,” and “*staphylococcus aureus* infection.” IPR enrichment analysis revealed that the DEPs were predominantly enriched in domains such as “ankyrin repeat-containing domain” and “globin” (Figure 4D). Subcellular localization prediction indicated that the majority of proteins were localized in the nucleus (21.79%), cytoplasm (16.67%), and extracellular space (14.10%) (Figure 4E).

**Table 3:** Top 10 up-regulated differentially expressed proteins of PT_CTX compared to C_CTX.

Protein accession	Protein description	Gene name	Fold change	Regulation type
V9HWA4	Betaine-homocysteine methyltransferase, isoform CRA_a	HEL-S-61p	330.7835694	Up
A0A1S5UZ39	Hemoglobin subunit alpha	HBA2	28.93313364	Up
A0N071	Delta globin	HBD	19.66367327	Up
P68871	Hemoglobin subunit beta	HBB	15.63303839	Up
P55084	Trifunctional enzyme subunit beta, mitochondrial	HADHB	15.40451727	Up
A0A5E4	Uncharacterized protein		12.82069667	Up
B4E1B2	Beta-1 metal-binding globulin		10.12237847	Up
Q9Y4P8	WD repeat domain phosphoinositide-interacting protein 2	WIPI2	6.891980639	Up
P02787	Serotransferrin	TF	6.246464834	Up
G4V2I9	Anion exchange protein		5.466163838	Up

PT_CTX, patient’s cortex sample; C_CTX, controls’ cortex sample.

**Table 4:** Top 10 down-regulated differentially expressed proteins of PT_CTX compared to C_CTX.

Protein accession	Protein description	Gene name	Fold change	Regulation type
P04350	Tubulin beta-4A chain	TUBB4A	41.10835694	Down
V9HW72	Epididymis secretory sperm binding protein	HEL-S-94n	19.03117358	Down
A8K4W8	cDNA FLJ77917, highly similar to *Homo sapiens* ubiquitin-conjugating enzyme E2L 3 (UBE2L3), transcript variant 1, mRNA		11.74082132	Down
D9YZV5	Tropomyosin 1 (Alpha) isoform 4	TPM1	10.84102669	Down
A0A2Z6ATB6	Drebrin A	DBN1	9.528770020	Down
V9HWI1	Epididymis secretory protein Li 10	HEL-S-10	8.216708320	Down
Q969H8	Myeloid-derived growth factor	MYDGF	7.457687523	Down
B4DUQ1	Heterogeneous nuclear ribonucleoprotein K		7.031921796	Down
A0A384NPU2	Epididymis secretory sperm binding protein	HINT1	6.613715016	Down
A0A024R9H2	2-iminobutanoate/2-iminopropanoate deaminase	HRSP12	6.330758238	Down

PT_CTX, patient’s cortex sample; C_CTX, controls’ cortex sample.

The PPI network analysis highlighted enriched DEP clusters primarily including “regulation of kinase activity,” “positive regulation of unsaturated fatty acid biosynthetic process,” “cGMP-PKG signaling pathway,” “transport of small molecules,” “regulation of small molecule metabolic process,” and others (Figure 4F). Six highly interconnected clusters were further identified as PPI networks (Supplementary Figure S2).

### Proteomic analysis of the nucleus of the patient’s lens versus the controls’ lens

**Figure 5:**
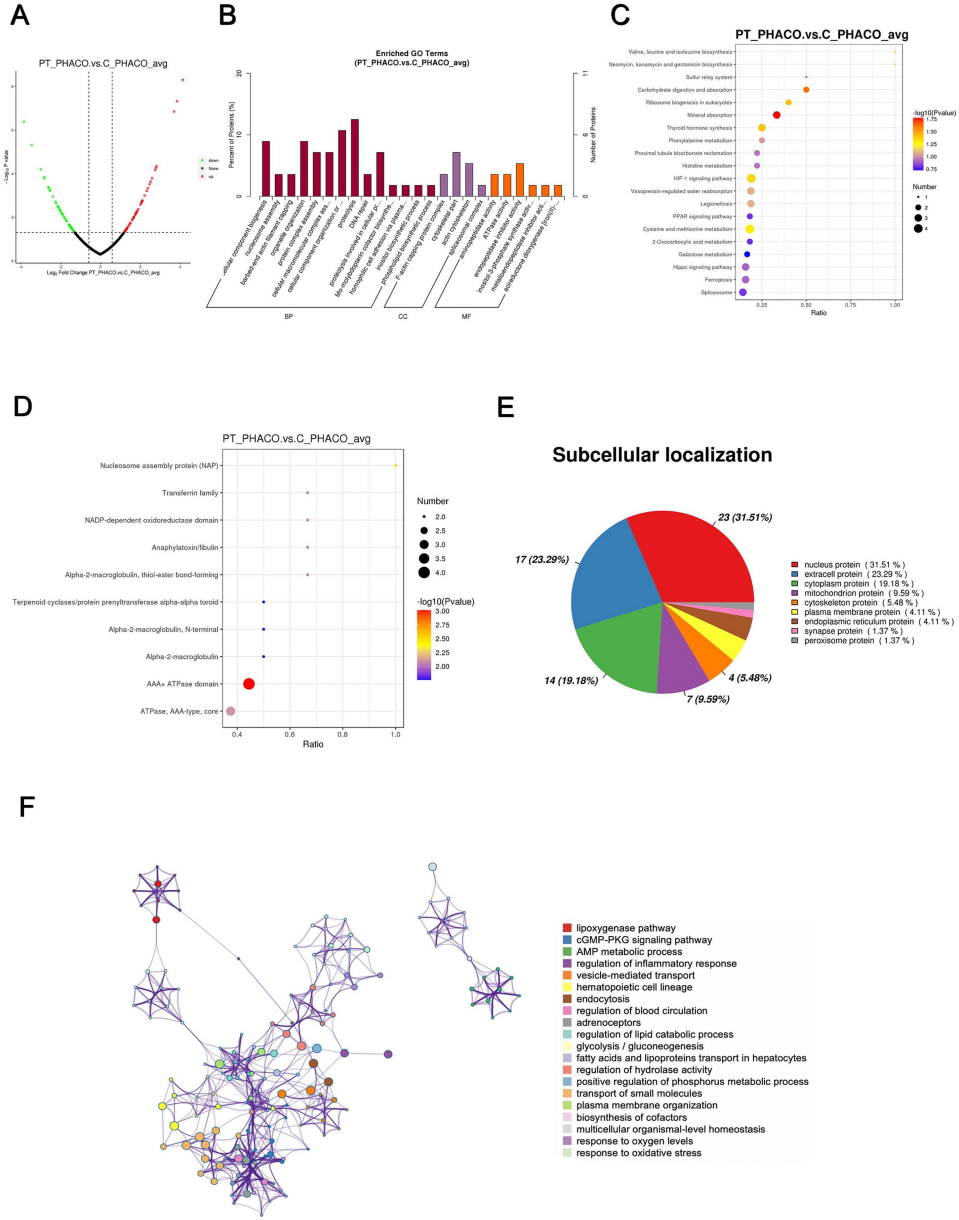
Quantitative proteomics analysis of DEPs between the patient’s and the controls’ phacoemulsification collect fluid. PT_PHACO, patient’s phacoemulsification collect fluid; C_PHACO, controls’ phacoemulsification collect fluid. **(A)** Volcano plot of DEPs. **(B)** GO classification of the DEPs between PT_PHACO and C_PHACO in terms of BP, CC, and MF. **(C)** Enrichment of KEGG pathways between PT_PHACO and C_PHACO. **(D)** IPR functional analysis of the DEPs between PT_PHACO and C_PHACO. **(E)** Subcellular localization of the DEPs between PT_PHACO and C_PHACO. **(F)** PPI network of DEPs between PT_PHACO and C_PHACO.

Ninety-five DEPs, comprising 51 upregulated and 44 downregulated proteins, were identified in the patient’s nucleus compared to controls, as illustrated in the volcano plot (Figure 5A). The top-10 upregulated and downregulated DEPs are listed in Tables 5, 6. GO functional classification (Figure 5B) indicated that DEPs were categorized into 24 GO terms, including 14 BPs, four CCs, and six MFs. Notably, “proteolysis,” “cellular component organization or biogenesis,” “cellular component biogenesis,” and “organelle organization” were the top four terms in the BPs. “Cytoskeleton” and “endopeptidase inhibitor activity” emerged as the most abundant terms in the CCs and MFs, respectively. The top 10 KEGG categories (Figure 5C) identified enriched pathways such as “mineral absorption,” “HIF-1 signaling pathway,” and “thyroid hormone synthesis.” IPR enrichment analysis revealed that the DEPs were predominantly enriched in the “AAA + ATPase domain” (Figure 5D). Subcellular localization prediction indicated that most proteins were located in the nucleus (31.51%), extracellular space (23.29%), and cytoplasm (19.18%) (Figure 5E).

**Table 5:** Top 10 up-regulated differentially expressed proteins of PT_PHACO compared to C_PHACO.

Protein accession	Protein description	Gene name	Fold change	Regulation type
B4E1B2	Beta-1 metal-binding globulin		17.39580711	Up
P11844	Gamma-crystallin A	CRYGA	14.27401896	Up
A0A087WYF2	Branched-chain-amino-acid aminotransferase, cytosolic (Fragment)	BCAT1	12.91080101	Up
P10114	Ras-related protein Rap-2a	RAP2A	7.017675415	Up
P55209	Nucleosome assembly protein 1-like 1	NAP1L1	6.867133433	Up
V9GYU0	Mitochondrial import inner membrane translocase subunit Tim17-B (Fragment)	TIMM17B	6.673217514	Up
V9HWA4	Betaine-homocysteine methyltransferase, isoform CRA_a	HEL-S-61p	5.991424756	Up
P02787	Serotransferrin	TF	5.717989048	Up
A0A024RBH2	Cytoskeleton-associated protein 4, isoform CRA_c	CKAP4	5.510783494	Up
Q6PIL8	IGK@ protein	IGK@	5.202968423	Up

PT_PHACO, patient’s phacoemulsification collect fluid; C_PHACO, controls’ phacoemulsification collect fluid.

**Table 6:** Top 10 down-regulated differentially expressed proteins of PT_PHACO compared to C_PHACO.

Protein accession	Protein description	Gene name	Fold change	Regulation type
P62979	Ubiquitin-40S ribosomal protein S27a	RPS27A	14.38184746	Down
A0A024QZS1	Molybdopterin synthase catalytic subunit	MOCS2	10.94058697	Down
A0A024R8G3	Glutathione-independent PGD synthase	PTGDS	7.960877802	Down
P10745	Retinol-binding protein 3	RBP3	7.107372107	Down
Q59FB2	Chordin-like 1 variant (Fragment)		7.005476655	Down
B4DG62	Hexokinase		6.118062159	Down
Q9GZS3	WD repeat-containing protein 61	WDR61	6.053459288	Down
B4DN40	cDNA FLJ54368, highly similar to Phosphoglucomutase-2		5.840435552	Down
Q9BV57	1,2-dihydroxy-3-keto-5-methylthiopentene dioxygenase	ADI1	5.789434575	Down
Q14019	Coactosin-like protein	COTL1	5.232246273	Down

PT_PHACO, patient’s phacoemulsification collect fluid; C_PHACO, controls’ phacoemulsification collect fluid.

The PPI network analysis revealed enriched DEP clusters primarily including “transport of small molecules,” “regulation of hydrolase activity,” “hematopoietic cell lineage,” and others (Figure 5F). Nine highly interconnected clusters were further identified as PPI networks (Supplementary Figure S3).

### Common DEPs in the anterior capsule, cortex, and nucleus of the patient’s lens versus controls

To elucidate the shared pathological mechanisms underlying lens opacification across each component of the lens, five commonly identified DEPs, A0A024R3E3 (APOA1), A0A140VKF3, P02787 (TF), Q6PIL8, and V9HWA9, within the anterior capsule, cortex, and nucleus of the patient’s lens were subjected to further analysis in comparison to controls (Table 7; Supplementary Figure S4). Notably, while all five DEPs exhibited downregulation in PT-CAP relative to C-CAP, they demonstrated upregulation in the cortex and nucleus of the patient’s lens compared to controls, with the exception of V9HWA9.

**Table 7:** Common differentially expressed proteins in the patient’s lens compared to the controls’.

Protein accession	Protein description	Gene name	Fold change (PT_CAP vs C_CAP)	Fold change (PT_CTX vs C_CTX)	Fold change (PT_PHACO vs C_PHACO)
A0A024R3E3	Apolipoprotein A-I, isoform CRA_a	APOA1	0.1989984947	4.989034771	3.506428364
A0A140VKF3	Testis tissue sperm-binding protein Li 70n		0.07342890159	3.641705978	4.144175186
P02787	Serotransferrin	TF	0.1060563140	6.246464834	5.717989048
Q6PIL8	IGK@ protein	IGK@	0.2090935418	2.412858553	5.202968423
V9HWA9	C3-beta-c	HEL-S-62p	0.2124909391	3.576505680	0.2614540740

## Discussion

This study unveiled the first documented instance of a toxic cataract induced by the inoculation of toad venom. Through proteomic sequencing and bioinformatics analyses, the lens sample extracted from the affected eye revealed a multitude of DEPs in comparison to the control group afflicted with non-toxic cataracts. Notably, the study identified five DEPs commonly expressed across the lens’s anterior capsule, cortex, and nucleus.

Venom ophthalmia, the ocular inoculation of venom, has been reported in association with a variety of animals, including snakes (5–9), toads (1–4), hornets (10–13), corals (14, 15), jellyfish, and anemones (16, 17). Rare instances involve spiders (18), caterpillars (19), moths (19), and walking sticks (20). The typical ocular manifestations resulting from these venoms include blepharitis, conjunctivitis, keratitis, corneal endothelial dysfunction, periorbital edema, iritis, or uveitis (5–10, 12, 14, 18, 20). More severe cases can lead to corneal erosions, ulceration, perforation, corneal decompensation, corneal and conjunctival neovascularization, cycloplegia, mydriasis, iris atrophy, ocular hypertonia or hypotonia, cataract, lens subluxation, lens abscess, optic neuropathy, and branch retinal artery occlusion (1, 2, 11–13, 15, 16). Specifically, ocular clinical manifestations from toad venom exposure may include chemosis, conjunctivitis, keratitis, stromal corneal edema with Descemet folds, corneal dysfunction, and ocular hypotonia (1–3). Very rarely, systemic reactions, including hypertension, bradycardia, and constriction of renal, mesenteric, and celiac arteries, may occur due to mucosal absorption of toad venom (4). Prior to this study, cases of cataracts caused by toad venom have not been reported.

Previous studies have established that toad venom primarily contains noxious substances such as bufadienolides (including bufofagins and bufotoxins with digitalis-like properties), bufotenines (exhibiting serotonin-like effects), and lipophilic alkaloids. Bufadienolides, acting as cardioactive substances, exert digitalis-like effects by inhibiting the Na^+^/K^+^-ATPase pump (21). This pump’s proper function is integral to the health of corneal endothelial cells, the ciliary body, and the iris, thereby playing a vital role in maintaining corneal transparency and regulating aqueous humor secretion (22, 23). Due to their highly lipophilic nature, bufadienolides can readily penetrate corneal layers, even with intact epithelium (24), potentially leading to corneal dysfunction and ocular hypotony. Furthermore, research suggests that systemic digoxin toxicity can also manifest as corneal edema, Descemet’s folds, and decreased intraocular pressure (24), thus reinforcing the previously described mechanisms. Despite the well-documented effects of toad venom, whether it induces cataracts through these mechanisms remains to be elucidated.

The present study conducted proteomic sequencing and bioinformatics analysis of lens specimens from a patient with cataracts induced by toad venom toxicity. A set of DEPs was identified in the patient’s lens compared to the control group. Notably, five DEPs were found to be commonly expressed across the anterior capsule, cortex, and nucleus of the lens: A0A024R3E3 (APOA1), A0A140VKF3, P02787 (TF), Q6PIL8, and V9HWA9. Previous reports have demonstrated that incubation of erythrocyte membranes with APOA1 results in decreased Na^+^/K^+^-ATPase activity (25). Additionally, research focusing on bufalin, one of the active constituents of toad venom and a member of cardiac steroids (CSs), confirmed the involvement of the Na^+^/K^+^-ATPase signalosome in the cytotoxicity of CSs. This research suggests that Apolipoprotein E regulates the sensitivity of cells to CSs by influencing the formation and function of the Na^+^/K^+^-ATPase signalosome (26). Another study highlights that CSs may cause the retention of TF in the early endosome. It is also suggested that CSs can increase acidification and the retention of recycled membrane within the early endosome or alter the sorting of the TF sensor to a more acidic compartment, such as the late endosome or lysosome (27).

In addition to the commonly identified DEPs in the anterior capsule, cortex, and nucleus of the lens, the present study unveiled several DEPs within each group’s top 10 rankings, which were associated with the functionality of Na^+^/K^+^-ATPase. Notably, the top three up-regulated DEPs in PT_CAP compared to C_CAP were MYH6, MYL2, and MYL3, as detailed in Table 1. It has been elucidated in prior research that myosin phosphatase (MP) interacts with Na^+^/K^+^-ATPase, thereby catalyzing the dephosphorylation of its inhibitory phosphorylation sites, thus designating this protein as a novel MP substrate (28). Furthermore, STAT1 demonstrates a notable upregulation in PT_CAP in comparison to C_CAP (Table 1). Studies underscore that interferon-gamma (IFN-g) significantly mitigates Na^+^/K^+^-ATPase activity through a cascade of transduction mechanisms set in motion by IFN-g, entailing the activation of PKC downstream of STAT1 phosphorylation, alongside the Raf-1, MEK, ERK2, and p38 MAPK pathways, in a complex sequence of events (29).

Ankyrin-1 (ANK1) was markedly downregulated in PT_CAP compared to C_CAP (Table 2). ANK1 exhibits a direct and high-affinity binding to recombinant peptides of the α1 subunit of Na^+^/K^+^-ATPase (30, 31). Ankyrin B (AnkB) functions as an adaptor protein, orchestrating the assembly of Na^+^/K^+^-ATPase and Na^+^/Ca^2+^ exchanger within the AnkB macromolecular complex (32). Mutations occurring within the established interaction motifs of Na^+^/K^+^-ATPase with AnkB and caveolin-1 are anticipated to perturb plasma membrane targeting, localization patterns, and the diffusion behavior of the enzyme (33).

Tropomyosin 1 (TPM1) displayed a significant downregulation in PT_CTX compared to C_CTX, as delineated in Table 4. Ouabain, a cardiac glycoside, exerts inhibitory effects on Na^+^/K^+^-ATPase, with its sensitivity being heightened by tropomyosin and calcium (34, 35). Moreover, hexokinase manifested a significant downregulation in PT_PHACO compared to C_PHACO, as indicated in Table 6. Investigations have elucidated that hexokinase interacts with ATP1A4, the testis-specific isoform of Na^+^/K^+^-ATPase, within a non-raft interactome (36).

Na^+^/K^+^-ATPase assumes a pivotal role in the maintenance of circulatory sodium homeostasis and regulation of hydrostatic pressure within the lens, primarily achieved by expelling Na^+^ ions into the extracellular space against their concentration gradient, thereby facilitating ionic flux to the equator (37). Notably, the activity of Na^+^/K^+^-ATPase exhibits variance between the two distinct cell types constituting the lens, namely epithelial cells and fibers, with epithelial cells displaying a higher specific activity compared to fibers. This discrepancy likely contributes to the observed differential expression of Na^+^/K^+^-ATPase-related DEPs across various lens sections.

Although the precise mechanisms await comprehensive elucidation, a robust correlation exists between aberrant lens sodium accumulation and cortical opacification observed in age-related human cataracts. Extant studies have implicated processes such as oxidation and glycation within aging lens fibers in diminishing Na^+^/K^+^-ATPase activity (38). Furthermore, experimental investigations in mice have demonstrated that inhibition of Na^+^/K^+^-ATPase pump activity can precipitate diverse biological and pathological phenomena, including cataractogenesis (39). Thus, it is plausible to infer that toad venom-induced cataracts may stem from Na^+^/K^+^-ATPase activity inhibition, perturbing ionic homeostasis within the lens. PPI network analysis of the DEPs in PT_CAP underscored their predominant association with functions such as “transportation of small molecules,” “regulation of metal ion transport,” and “import into cell,” thereby lending further credence to this hypothesis. However, a deeper comprehension of these mechanisms necessitates further investigation. While surgical intervention currently stands as the primary therapeutic modality for cataracts (40), the insights gleaned from these findings held promise for informing the prevention and management of toxic cataracts engendered by toad venom in future therapeutic endeavors.

This study is subject to certain limitations, primarily stemming from constraints related to the availability of patient specimens. Firstly, due to the rarity of such cases, the study group comprised only a single patient’s lens sample. Secondly, owing to the limited volume of protein specimens, conducting further validation of the DEPs was not feasible. Thirdly, concerning the impact of long-term sample storage on the accuracy of test results, lens samples from age-related cataract patients who underwent surgery on the same day were included as the control group, rather than congenital cataract patients of similar age as the patient in the present case, since the latter are relatively rare. Additionally, as the patient did not promptly seek medical attention following exposure to the toad venom, it remained impossible to ascertain the presence of any transient conditions such as low intraocular pressure, keratitis, or uveitis at the time of examination.

In summary, this study presented a unique case of ocular toad venom inoculation, representing the first documented instance of toxic cataract induced by inadvertent exposure to toad venom splash. Furthermore, proteomic sequencing was conducted on the lens specimen obtained from the patient, aimed at unraveling the underlying pathological mechanisms. The findings suggested a potential association between the development of cataract and alterations in Na^+^/K^+^-ATPase activity. This research offered valuable insights and might serve as a guiding framework for the clinical management of ocular toad venom inoculation in the future.

## Data Availability

The datasets presented in this study can be found in online repositories. The names of the repository/repositories and accession number(s) can be found below: http://www.proteomexchange.org/, PXD058328.
