# Supplementary material for: Ocular inoculation of toad venom: toxic cataract and proteomic profiling
Source: Front Med (Lausanne). 2025 Jan 14;11:1537770. doi: 10.3389/fmed.2024.1537770 (PMC11772289; doi:10.3389/fmed.2024.1537770)
Supplement: Supplementary file 1 [file Data_Sheet_1.PDF]

Supplementary Materials

Supplementary Materials and Methods

Clinical Data Collection

Standard preoperative and postoperative assessments encompassed evaluations of uncorrected distance visual acuity (UDVA), best-corrected distance visual acuity (BCDVA), slit-lamp biomicroscopy with photography (SL-D701, Topcon), dilated fundoscopy with photography (CR-2 Plus AF, Canon Inc.), intraocular pressure (IOP) measurement (NT-510, Nidek Co., Ltd.), B-mode ultrasonography (Compact Touch, Quantel Medical), corneal topography via Scheimpflug imaging (Pentacam, Oculus Optikgeräte GmbH), optical biometry (IOLMaster 700, Carl Zeiss Meditec. Inc), and ultrasound pachymetry and biometry (Cinescan, Quantel Medical).

4D Label-Free Quantitative Proteomic Analysis

Total Protein Extraction

Individual samples were pulverized in liquid nitrogen and subsequently lysed using PASP lysis buffer. The lysates underwent 5 minutes of ultrasonication on ice followed by centrifugation at 12,000 g for 15 minutes at 4°C. Subsequently, 10 mM DTT was added to the supernatant. After incubating at 56°C for 1 hour, the supernatant was alkylated with sufficient IAM for 1 hour at room temperature in the dark. The specimens were thoroughly mixed with a fourfold volume of pre-cooled acetone by vortexing and then incubated at -20°C for at least 2 hours. The precipitate was collected after centrifugation at 12,000 g for

15 minutes at 4°C. After washing with 1 mL of cold acetone, the pellet was dissolved in Dissolution Buffer (DB buffer).

Protein Quality Assessment

A bovine serum albumin (BSA) standard protein solution was prepared according to the Bradford protein quantitative kit instructions, with concentrations ranging from 0 to 0.5 g/L. BSA standard protein solutions and sample solutions with various dilution factors were added to a 96-well plate, each in a volume of 20 µL. This process was repeated three times for each gradient. Next, 180 µL of G250 dye solution was swiftly added to each well and left at room temperature for 5 minutes before measuring absorbance at 595 nm. A standard curve was constructed using the absorbance of the standard protein solution, facilitating the calculation of sample protein concentrations. Additionally, 20 µg of the protein sample was loaded onto a 12% SDS-PAGE gel electrophoresis apparatus. Electrophoresis was conducted at 80 V for 20 minutes for the concentrated gel and at 120 V for 90 minutes for the separation gel. Subsequently, the gel was stained with Coomassie Brilliant Blue R-250 and decolorized until bands were clearly visualized.

Trypsin Treatment

Each protein sample was adjusted to a volume of 100 µL with DB lysis buffer (8 M Urea, 100 mM TEAB, pH 8.5). Trypsin and 100 mM TEAB buffer were added, and the sample was thoroughly mixed and then digested at 37°C for 4 hours. Subsequently, additional trypsin and $CaCl_2$ were added, and the digestion process continued overnight. Following digestion, formic acid was

mixed with the sample to adjust the pH to below 3, and the mixture was then centrifuged at 12,000 g for 5 minutes at room temperature. The supernatant was slowly loaded onto a C18 desalting column, washed three times with washing buffer (0.1% formic acid, 3% acetonitrile), and subsequently eluted with elution buffer (0.1% formic acid, 70% acetonitrile). The eluates from each sample were collected and lyophilized.

Liquid Chromatography-Tandem Mass Spectrometry (LC-MS/MS) Analysis

Ultra-high performance liquid chromatography (UHPLC)-MS/MS analyses were conducted using a nanoElute UHPLC system (Bruker, Germany) coupled with a tims TOF pro2 mass spectrometer (Bruker, Germany) at Novogene Co., Ltd. (Beijing, China). Initially, mobile phase solutions A (100% water, 0.1% formic acid) and B (100% acetonitrile, 0.1% formic acid) were prepared. The lyophilized powder was dissolved in 10 μL of solution A, centrifuged at 14,000 g for 20 minutes at 4°C, and 200 ng of the supernatant was injected into the liquid chromatography-mass spectrometry system for detection. The UHPLC model utilized was nanoElute with nano-upgraded features, and the analytical column employed was a custom-made analytical column (15 cm × 100 μm, 1.9 μm). The elution conditions for liquid chromatography are detailed in Table S1.

The tims TOF pro2 mass spectrometer utilized a Captive Spray ion source, with a spray voltage set to 4.5 kV. The full scan range of mass was from m/z 100 to 1,700, with a Ramp time of 100 ms. The 1/K0 parameter was set from 0.6 to 1.6 V·s/cm2, and the Lock Duty Cycle was set to 100%. Parameters for

PASEF were configured as follows: 10 MS/MS scans (with a total cycle time of 1.17 sec), an ionic strength threshold of 2,500, and a scheduling target intensity of 20,000. The raw data from MS detection were saved with a ".d" extension.

Data Analysis

Identification and Quantification of Proteins

The acquired spectra were interrogated against the Homo sapiens UniProt database (release date: July 15, 2021; containing 202,195 sequences) using the MaxQuant search engine (Bruker, Tims). The search parameters were configured as follows: a mass tolerance of 20 ppm for precursor ions and 0.05 Da for product ions. Carbamidomethyl was designated as a fixed modification, while oxidation of methionine and acetylation were set as dynamic and N-terminal modifications, respectively. Up to 2 missed cleavage sites were permitted.

Protein quantitation outcomes underwent statistical scrutiny via T-test analysis. Proteins exhibiting significant quantitative disparities between experimental and control groups, with a P-value ≤ 0.05 and an absolute log2(fold change) value ≥ 1.5, were designated as differentially expressed proteins (DEPs).

Functional Analysis of Proteins and DEPs

Gene Ontology (GO) and InterPro (IPR) functional assessments were performed utilizing the InterProScan program against a non-redundant protein database, incorporating Pfam, PRINTS, ProDom, SMART, ProSite, and PANTHER databases. Additionally, the Clusters of Orthologous Groups (COG)

and Kyoto Encyclopedia of Genes and Genomes (KEGG) databases were leveraged for protein family and pathway analyses. DEPs were subjected to Volcano plot analysis, cluster heat map analysis, and enrichment analysis encompassing GO, IPR, and KEGG categories. The putative protein-protein interaction (PPI) network was forecasted and scrutinized using the Metascape server (https://metascape.org).

Table S1 NanoElute Liquid chromatography elution gradient table

Time (min)	flow rate (mL/min)	mobile phase A (%)	mobile phase B (%)
0	900	98	2
5	900	96	4
10	300	94	6
35	300	78	22
45	300	65	35
47.5	300	20	80
50	300	20	80

Table S2 General information of the DEPs in the anterior capsule, cortex, and nucleus of the patient's lens compared to controls'

Group	Number of DEPs	Number of up-regulated DEPs	Number of up-regulated DEPs
PT_CAP vs C_CAP	204	92	112
PT_CTX vs C_CTX	109	53	56
PT_PAHCO vs C_PHACO	95	51	44

Supplementary Figure Captions

Figure S1. MCODE clusters depicting the DEPs between PT_CAP and C_CAP.

Figure S2. MCODE clusters illustrating the DEPs between PT_CTX and C_CTX.

Figure S3. MCODE clusters displaying the DEPs between PT_PHACO and C_PHACO.

Figure S4. Commonly identified DEPs in each lens component between patients and controls. (A) Venn diagram illustrating the comparison of DEPs among PT_CAP vs. C_CAP, PT_CTX vs. C_CTX, and PT_PHACO vs. C_PHACO. (B) Fold change analysis of the common DEPs between each pair: PT_CAP and C_CAP, PT_CTX and C_CTX, and PT_PHACO and C_PHACO.

Figure S1

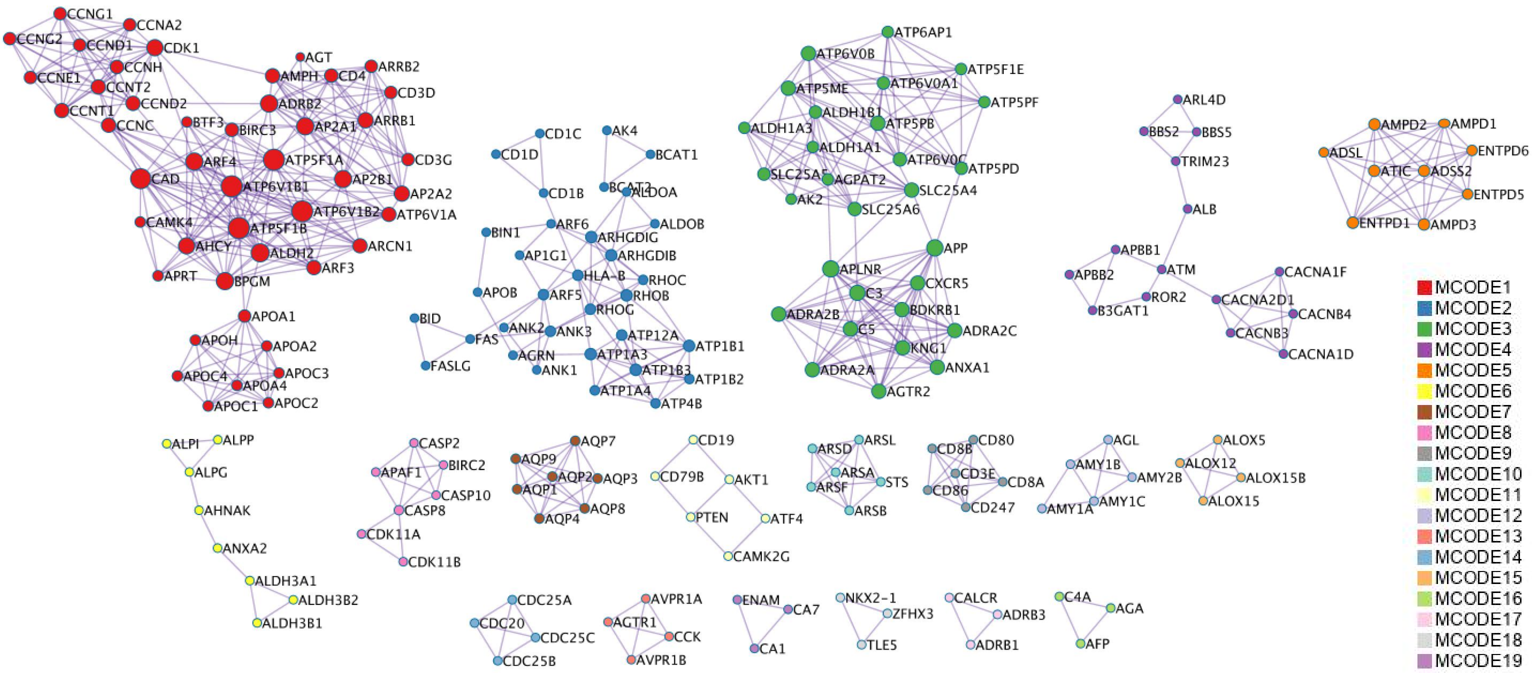
MCODE1
MCODE2
MCODE3
MCODE4
MCODE5
MCODE6
MCODE7
MCODE8
MCODE9
MCODE10
MCODE11
MCODE12
MCODE13
MCODE14
MCODE15
MCODE16
MCODE17
MCODE18
MCODE19

Figure S2

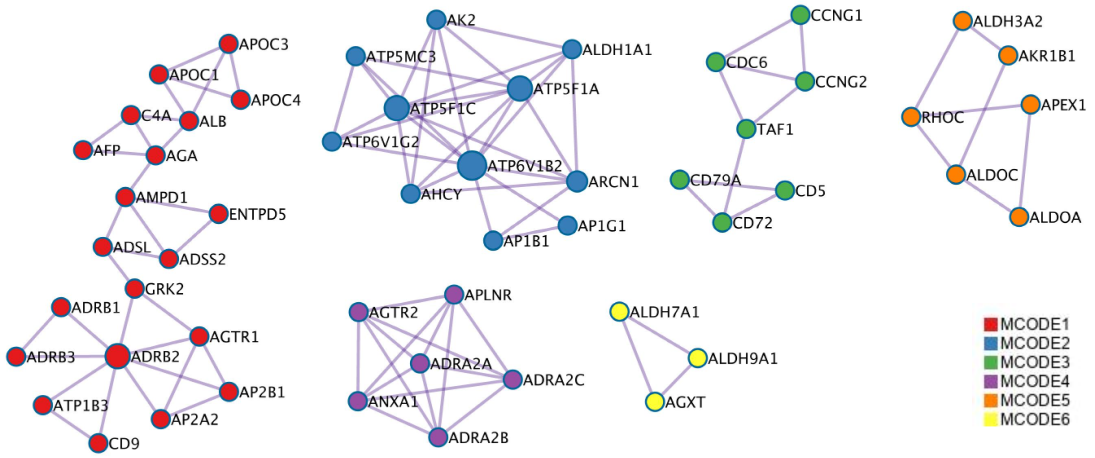
APOC3
APOC1
APOC4
C4A
ALB
AFP
AGA
AMPD1
ENTPD5
ADSL
ADSS2
GRK2
ADRB1
AGTR1
ADRB3
ADRB2
AP2B1
ATP1B3
AP2A2
CD9
AK2
ATP5MC3
ALDH1A1
ATP5F1A
ATP5F1C
ATP6V1G2
ATP6V1B2
ARCN1
AHCY
AP1G1
AP1B1
CCNG1
CDC6
CCNG2
TAF1
CD79A
CD5
CD72
ALDH3A2
AKR1B1
RHOC
APEX1
ALDOC
ALDOA
APLNR
AGTR2
ADRA2A
ADRA2C
ANXA1
ADRA2B
ALDH7A1
ALDH9A1
AGXT
MCODE1
MCODE2
MCODE3
MCODE4
MCODE5
MCODE6

Figure S3

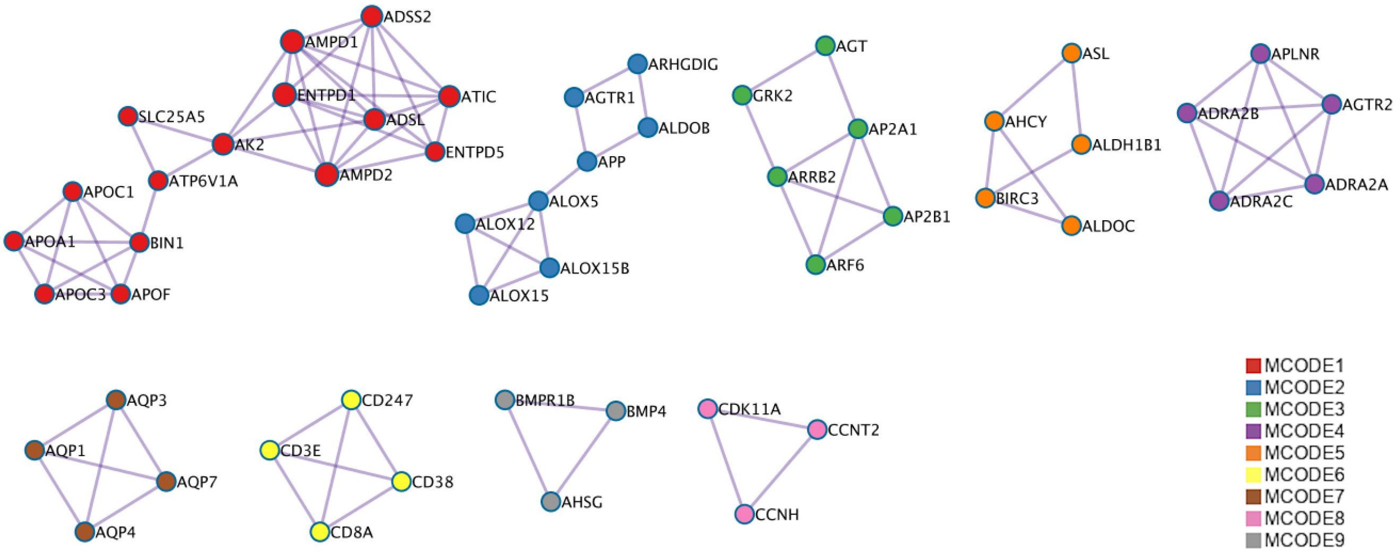

ADSS2
AMPD1
ENTPD1
ADSL
ATIC
ENTPD5
AMPD2
AK2
SLC25A5
ATP6V1A
APOC1
APOA1
BIN1
APOC3
APOF
ARHGDIG
AGTR1
ALDOB
APP
ALOX5
ALOX12
ALOX15B
ALOX15
AGT
GRK2
AP2A1
ARRB2
AP2B1
ARF6
ASL
AHCY
ALDH1B1
BIRC3
ALDOC
APLNR
ADRA2B
AGTR2
ADRA2A
ADRA2C
AQP3
AQP1
AQP7
AQP4
CD247
CD3E
CD38
CD8A
BMPR1B
BMP4
AHSG
CDK11A
CCNT2
CCNH
MCODE1
MCODE2
MCODE3
MCODE4
MCODE5
MCODE6
MCODE7
MCODE8
MCODE9

Figure S4

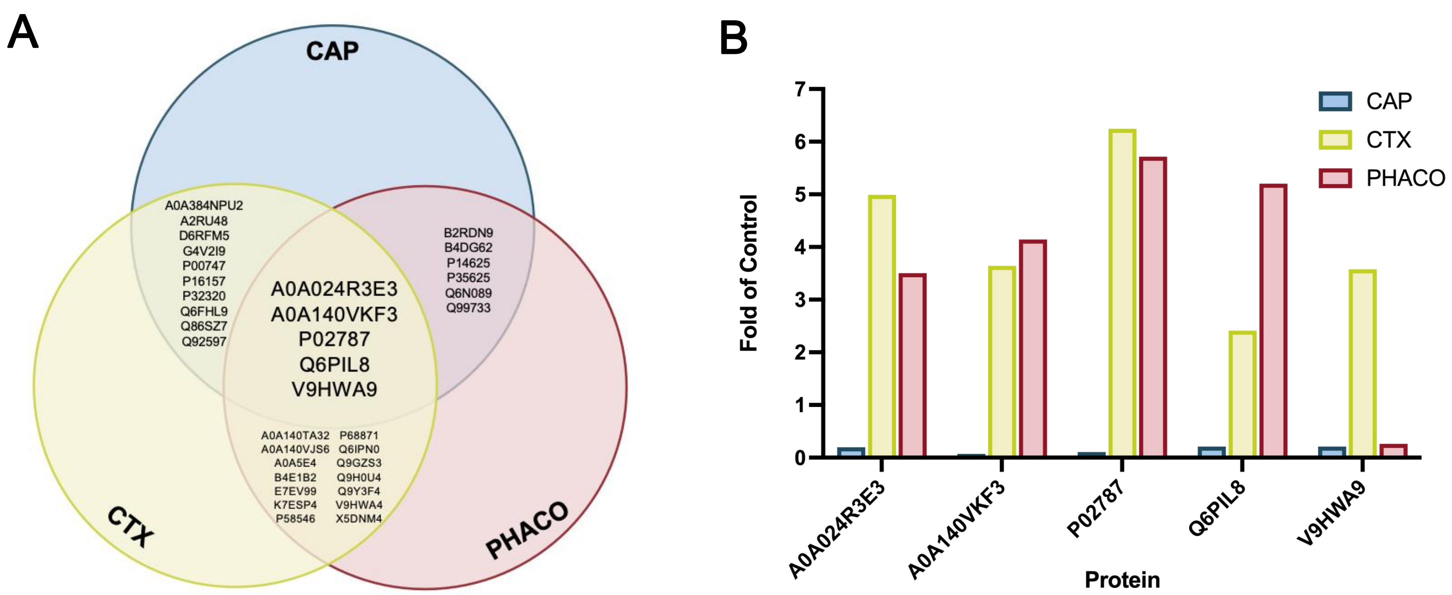
A
CAP
A0A384NPU2
A2RU48
D6RFM5
G4V2I9
P00747
P16157
P32320
Q6FHL9
Q86SZ7
Q92597
B2RDN9
B4DG62
P14625
P35625
Q6N089
Q99733
A0A024R3E3
A0A140VKF3
P02787
Q6PIL8
V9HWA9
A0A140TA32
A0A140VJS6
A0A5E4
B4E1B2
E7EV99
K7ESP4
P58546
P68871
Q6IPN0
Q9GZS3
Q9H0U4
Q9Y3F4
V9HWA4
X5DNM4
CTX
PHACO
B
Fold of Control
0
1
2
3
4
5
6
7
CAP
CTX
PHACO
A0A024R3E3
A0A140VKF3
P02787
Q6PIL8
V9HWA9
Protein
